# NTCU induced pre-malignant and malignant stages of lung squamous cell carcinoma in mice model

**DOI:** 10.1038/s41598-021-01988-8

**Published:** 2021-11-18

**Authors:** Muhammad Asyaari Zakaria, Nor Fadilah Rajab, Eng Wee Chua, Gayathri Thevi Selvarajah, Siti Fathiah Masre

**Affiliations:** 1 Centre for Toxicology and Health Risk Studies, Faculty of Health Sciences, Universiti Kebangsaan Malaysia, 50300 Kuala Lumpur, Malaysia; 2 Centre for Healthy Ageing and Wellness, Faculty of Health Sciences, Universiti Kebangsaan Malaysia, 50300 Kuala Lumpur, Malaysia; 3 Faculty of Pharmacy, Universiti Kebangsaan Malaysia, 50300 Kuala Lumpur, Malaysia; 4 Department of Veterinary Clinical Studies, Faculty of Veterinary Medicine, Universiti Putra Malaysia (UPM), 43400 Serdang, Malaysia

**Keywords:** Cancer models, Non-small-cell lung cancer

## Abstract

Mice have served as an excellent model to understand the etiology of lung cancer for years. However, data regarding dual-stage carcinogenesis of lung squamous cell carcinoma (SCC) remain elusive. Therefore, we aim to develop pre-malignant (PM) and malignant (M) lung SCC in vivo using N-nitroso-tris-chloroethylurea (NTCU). BALB/C mice were allotted into two main groups; PM and M groups which received treatment for 15 and 30 weeks, respectively. Then, the mice in each main group were allotted into three groups; control, vehicle, and cancer (n = 6), which received normal saline, 70% acetone, and 0.04 M NTCU by skin painting, respectively. Histopathologically, we discovered a mix of hyperplasia, metaplasia, and dysplasia lesions in the PM group and intracellular bridge; an SCC feature in the M group. The M group was positive for cytokeratin 5/6 protein which confirmed the lung SCC subtype. We also found significantly higher (*P* < 0.05) epithelium thickness in the cancer groups as compared to the vehicle and control groups at both the PM and M. Overall, this study discovered that NTCU is capable of developing PM and M lung SCC in mice model at appropriate weeks and the vehicle group was suggested to be adequate as control group for future research.

## Introduction

Lung cancer (LC) is one of the leading causes of cancer-related death worldwide with an estimated 1.8 million deaths in 2020^1^. Lung cancer is histologically classified into two types: non-small cell lung cancer (NSCLC) and small cell lung cancer (SCLC). The NSCLC subtype accounts for 85% of all lung cancer cases, which are further classified into three main subtypes: large cell lung cancer, adenocarcinoma, and squamous cell carcinoma (SCC). Due to their poor therapeutic responses^2^ and low survival rate^3^, lung SCC subtypes have received increased attention in the past decades. However, the etiology of lung SCC remains poorly understood. Lung SCC is a heterogeneous disease that progresses through multiple stages^4,5^. Moreover, the lung SCC subtype has a complex and distinct genetic background when compared to other NSCLC subtypes^6–9^, which may explain the limitations of current NSCLC therapy in treating lung SCC patients. Notably, most of the studies on NSCLC and lung SCC were conducted in vitro instead of in vivo or animal model^10^. Thus, there is an urgent need to establish well-characterized and reliable lung SCC animal models for pre-clinical studies that will provide novel insight into lung SCC biology in the future.

The use of mice in pre-clinical lung cancer studies has been shown to improve our understanding of disease pathogenesis and elucidate therapeutic options^11^. According to a growing number of studies, chemical-induced cancer in animals is the best model for understanding cancer etiology and carcinogenesis as it closely mimics chronic environmental carcinogen exposure^12^. In this regard, N-nitroso-tris-chloroethylurea (NTCU); a component of nitrosoalkylureas, has been shown to induce lung SCC in mice models with a high degree of similarity and comparable histology to human lung SCC^5,13–16^. According to Wang et al., NTCU can introduce the same sequence of lung SCC lesions seen in humans, namely ‘normal-hyperplasia-metaplasia-dysplasia-SCC’^5^. Moreover, RNA sequencing of lung SCC induced in the animal model using NTCU showed a high percentage of mutation similarity to human SCC^17^. As a result, this model closely resembles human SCC and has been proposed as a feasible reservoir to define SCC biology, which is still poorly understood.

However, the use of NTCU still possess some challenges due to the disparity in the ability of NTCU to induce lung SCC in different strains of mice. Notably, the disparity was also influenced by the dose, volume, and duration of NTCU treatment. Thus, the use of an optimal dose, volume, and duration of treatment, as well as the use of the appropriate strain of mice, is essential to achieve the desired outcome following the research objective. Moreover, the characterization of NTCU-induced lung SCC at the pre-malignant and malignant stages, which is critical for testing the best candidate drugs and preventive agents in pre-clinical studies, is still poorly understood. Therefore, the present study aims to develop the PM and M stages of lung SCC using NTCU and classify the dual-stage based on SCC lesions characteristics in BALB/c mice, which was categorized as an intermediate susceptibility strain to lung SCC carcinogen. The severity of the lesions is determined by referring to histopathology scores and measuring epithelium thickness. Lung SCC subtype of lung cancer was confirmed by positive immunohistochemistry for cytokeratin (CK) 5/6 protein, an SCC Biomarker.

## Results

### Specific pre-malignant and malignant stages of lung SCC subtype by NTCU

**Figure 1:**
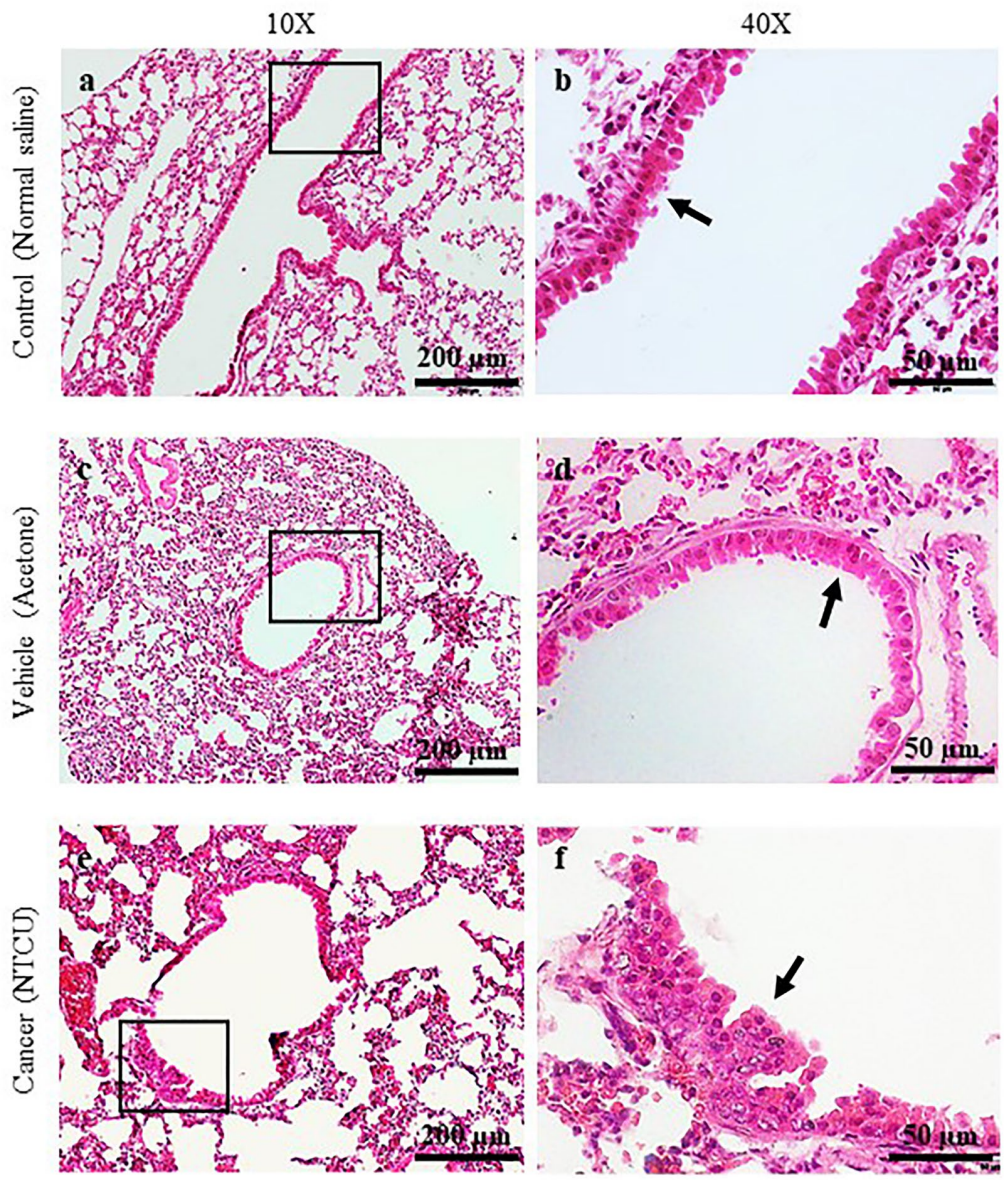
H&E Staining of the bronchial area after 15 weeks of treatment with normal saline (**a** and **b**), acetone (**c** and **d**), and NTCU (**e** and **f**). Arrows in (**b**) and (**d**) show normal bronchial epithelial cells. Arrow in **f** shows hyperplastic epithelial cells.

**Figure 2:**
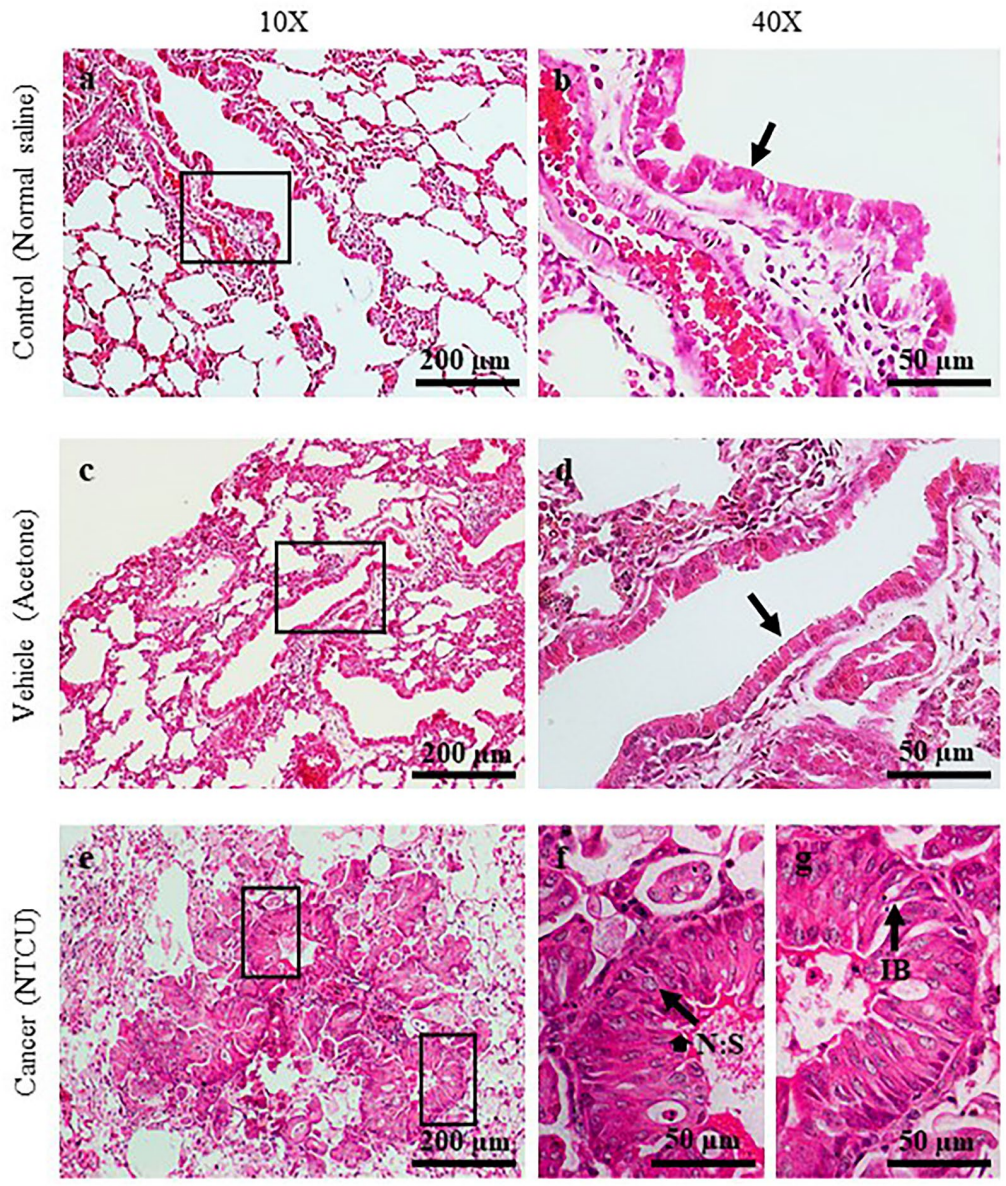
H&E Staining of the bronchial area after 30 weeks of treatment with normal saline (**a** and **b**), acetone (**c** and **d**), and NTCU (**e**, **f**, and **g**). Arrows in (**b**) and (**d**) show normal bronchial epithelial cells. Arrow in **f** shows cancer cells with a high nuclear to cytoplasm (N:S) ratio. Arrow in **g** shows intracellular bridge (IB).

H&E staining is a gold standard assay for determining tissue histology. In this study, SCC lesions were classified as pre-malignant (PM) and malignant (M) stages based on the layer of epithelial cells, the appearance of squamous cells that replaces epithelial cells, the nucleus to cytoplasm ratio, and the formation of intracellular bridges and/or keratin pearls. Figure 1 depicts the H&E staining at the bronchial area after 15 weeks of treatment with normal saline (a and b), acetone (c and d), and NTCU (e and f). Figure 2 shows the H&E staining at the bronchial area after 30 weeks of treatment with normal saline (a and b), acetone (c and d), and NTCU (e and f). Tissues were then scored based on the histology characteristics observed in three sections per mouse, and the score obtained for both treatment durations are shown in Tables 1 and 2.

**Table 1:** Histopathology score of lung tissue after 15 weeks of treatment.

Group	Histology characteristic	Stage of carcinogenesis	n	Score (Mean ± SEM)
Control	Normal	No	6	0
Vehicle	Normal	No	6	0
Cancer	Hyperplasia, Metaplasia, and Dysplasia	Early and Intermediate	6	2.00 ± 0.29*

*Significant difference (*P* < 0.05) between cancer with vehicle and control groups.

**Table 2:** Histopathology score of lung tissue after 30 weeks of treatment.

Group	Histology characteristic	Stage of carcinogenesis	n	Score (Mean ± SEM)
Control	Normal	No	6	0
Vehicle	Normal	No	6	0
Cancer	SCC	Final	6	4.00 ± 0.00*

*Significant difference (*P* < 0.05) between cancer with vehicle and control groups.

In general, mice that were induced with normal saline and acetone for 15 dan 30 weeks show normal bronchial epithelial cell characteristics (Score 0), as represented by a single layer of regular cell shape. This normal characteristic was pointed by arrows in Fig. 1b and d, and Fig. 2b and d at 40× magnification. Meanwhile, mice treated with NTCU for 15 weeks develop a mix of hyperplasia, metaplasia, and dysplasia lesions that can be categorized as PM. Notably, hyperplasia and metaplasia lesions were the most frequent type of lesions discovered, explaining the score of 2.00 ± 0.29. The hyperplasia lesion, presented as multiple and stratified layers of bronchial epithelial cells was pointed by an arrow in Fig. 1f at 40X magnification. Furthermore, mice induced with NTCU for 30 weeks show a mixture of SCC in situ and invasive SCC lesions (score 4), which can be categorized as M stage. The SCC lesions are indicated by arrows in Fig. 2f and g, which shows a cancer cell with a high nuclear to cytoplasm ratio and an intracellular bridge. The intracellular bridge is the distinguishing feature of SCC besides the keratin pearl. However, keratin pearl was not observed in this study.

NTCU-induced lung SCC in female BALB/c mice was found to be histopathologically similar to human lung SCC, suggesting the high reproducibility of NTCU in producing a comparable model of human lung SCC in mice. Table 3 describes the explanation for each lung SCC stage based on the histology characteristic discovered in this study.

**Table 3:** Description for each stage of lung SCC histology characteristics.

Stages	Characteristics
Normal	Single layer of columnar bronchial epithelial cells
Hyperplasia	** > **1 layer of columnar bronchial epithelial cells
Metaplasia	Columnar bronchial epithelial cells were replaced by squamous epithelial cells while maintaining hyperplasia characteristic
Dysplasia	A high degree of nuclear pleomorphism and horizontally orientated nuclei were observed at the upper epithelium. The cells also lose orderly differentiation, irregular in shape, and have a high nucleus to cytoplasm ratio through the entire thickened epithelium
SCC	The neoplastic cells become irregular in shape, increased nucleus to cytoplasm ratio, and some nuclei were observed as hyperchromatic. The cancer cells are capable of invading surrounding stroma by breaching the basement membrane

### Increased epithelium thickness in NTCU-induced pre-malignant and malignant stages

**Figure 3:**
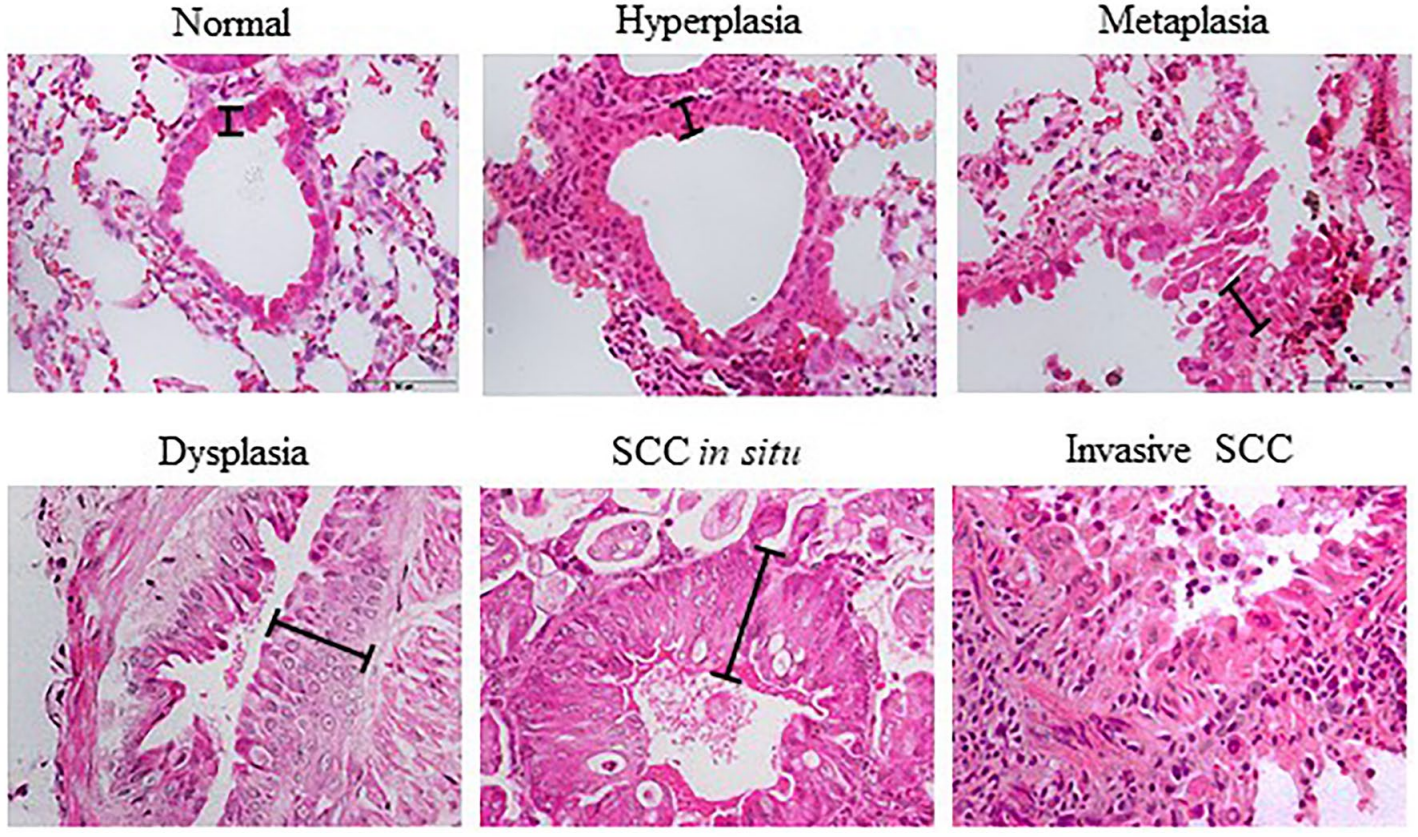
NTCU induces lung SCC through ‘normal-hyperplasia-metaplasia-dysplasia-SCC’ sequence in mice model. The lines show the length of epithelial cells measured for epithelium thickness analysis.

The thickness of the bronchial epithelium was measured from H&E staining and is shown in Fig. 3. At the PM stage, the epithelium thickness of the cancer group (20.72 ± 1.27 μm) was significantly higher (*P* < 0.05) than that of the control (11.05 ± 0.23 µm) and vehicle (11.53 ± 0.35 µm) groups. Similarly, at the M stage, the epithelium thickness of the cancer group (39.71 ± 2.27 µm) was found to be significantly higher (*P* < 0.05) than the control (11.54 ± 0.33 µm) and vehicle (10.86 ± 0.34 µm) groups. Moreover, we found that the bronchial epithelium thickness in the cancer group at the M stage was significantly higher (*P* < 0.05) than in the cancer group at the PM stage. Besides, there was no significant difference in epithelium thickness between control and vehicle from the same stage of carcinogenesis or between two different stages.

### Lung SCC subtype confirmation by cytokeratin 5/6 protein expression

**Figure 4:**
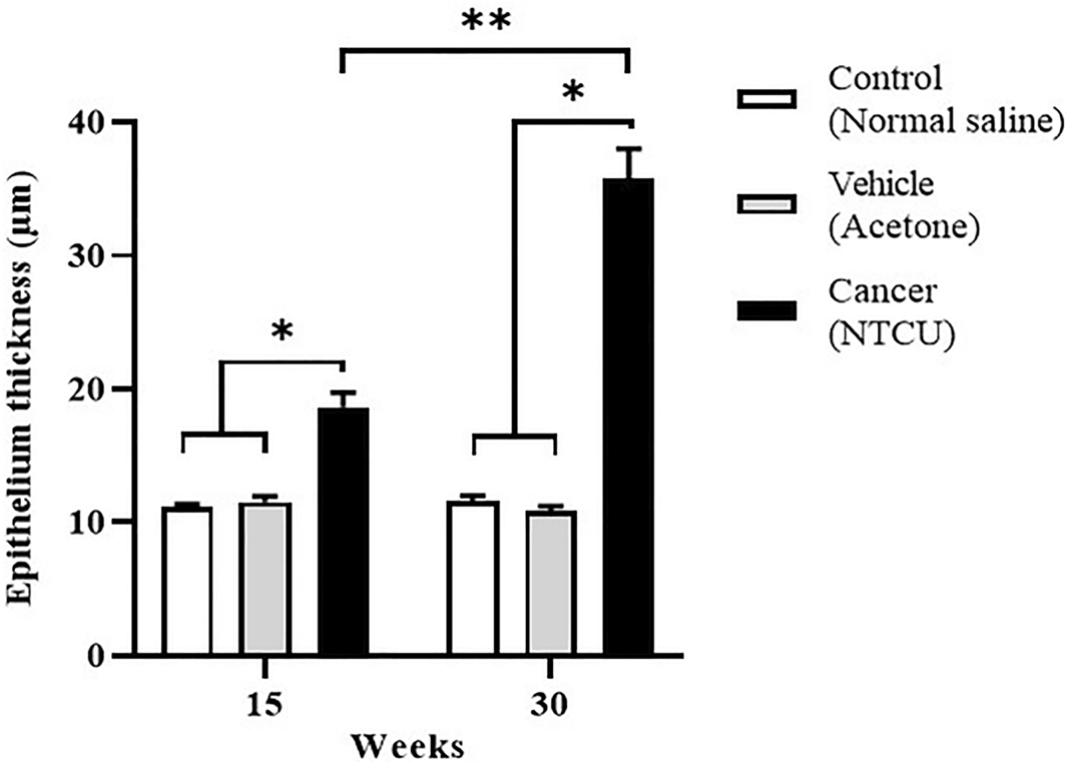
Epithelium thickness after 15 (pre-malignant stage) and 30 weeks (malignant stage) of treatment with normal saline, acetone, and NTCU. *Significant difference (*P* < 0.05) between groups in the same stage. **Significant difference between cancer groups from two different stages.

**Figure 5:**
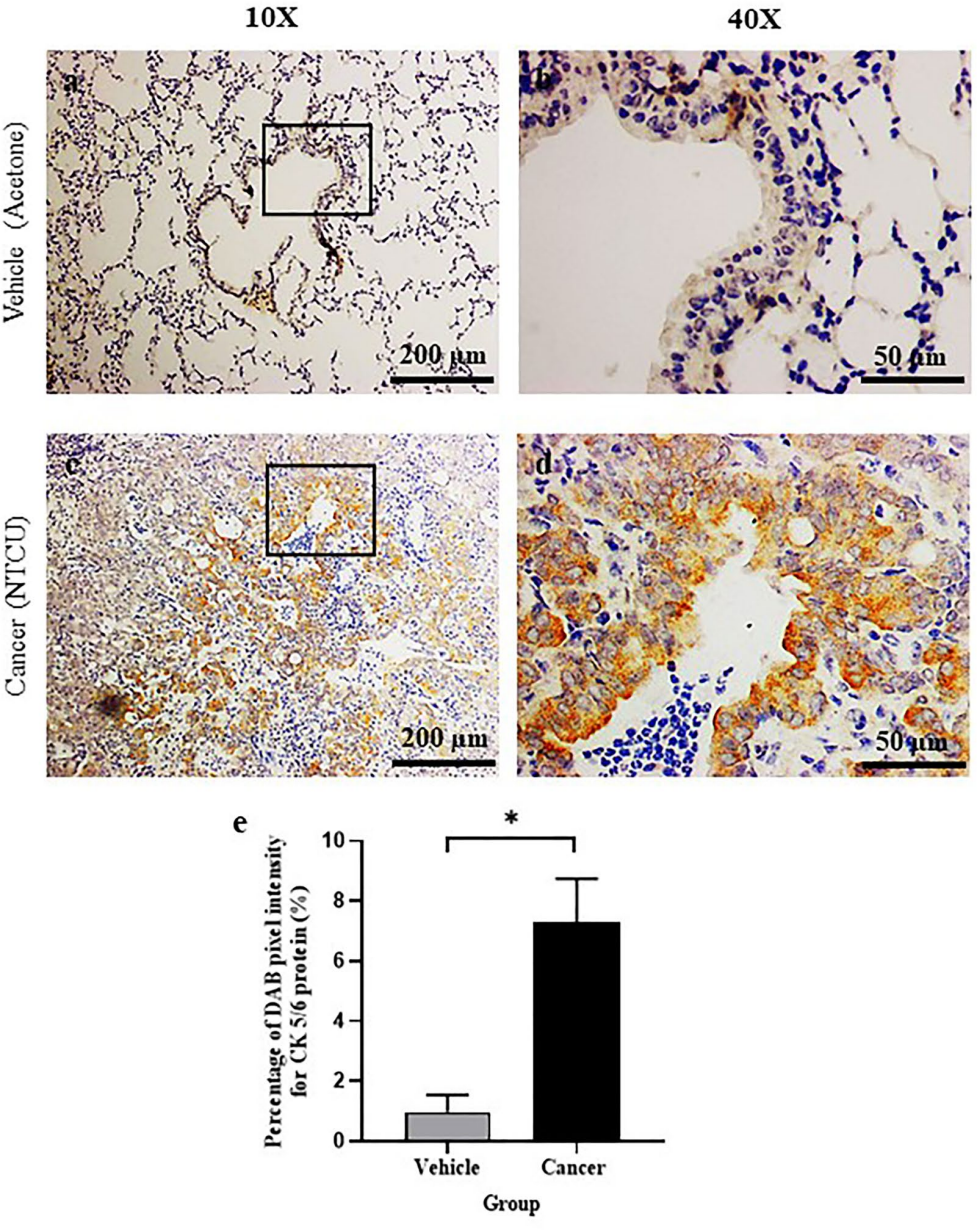
Cytokeratin (CK) 5/6 IHC Staining at the bronchial area after 30 weeks (malignant stage) of treatment with acetone (**a** and **b**) and NTCU (**c** and **d**). Brown staining showing positive immunoreaction of CK 5/6. (**e**) shows the percentage of DAB pixel intensity for CK 5/6 protein (%) at the bronchial area after 30 weeks (malignant stage) of treatment with acetone and NTCU. *Significant difference (*P* < 0.05) between vehicle and cancer group.

IHC staining for cytokeratin 5/6 (CK 5/6) protein, an SCC marker was determined around the bronchial tissue of the M stage. Based on the histological observations, the M cancer group from 30 weeks of NTCU treatment has SCC histological feature, and CK 5/6 protein expression was evaluated to confirm this subtype. As shown in Fig. 4, the cancer group shows positive brown DAB staining for CK 5/6 protein as compared to vehicle. Figure 5 shows a bar chart of DAB pixel intensity, which corresponds to quantitative CK 5/6 protein expression. Based on the bar chart, the cancer group had a significantly higher percentage of pixel intensity (*P* < 0.05) which was 6.36 ± 0.86% as compared to the vehicle group; 0.44 ± 0.18%.

## Discussion

Aside from human tumor xenografts and transgenic animals, chemically induced lung cancers in animal models are also widely used in the studies of lung cancer^12^. N-nitroso-tris-chloroethylurea (NTCU) is commonly used to induce lung SCC in mouse models, as it gives reproducible results^13–15,17–23^. NTCU is also easy to administer, requiring only skin painting as compared to benzo(α)pyrene which requires direct instillation into the trachea^24,25^. Once absorbed through the skin, NTCU acts as an alkylating agent, causing DNA mutation and cancer^26^. The effectiveness of NTCU in inducing lung SCC depends on the strain of mice, which can be classified into three categories: susceptible (e.g., A/J, NIH Swiss dan SWR/J), intermediate (e.g., BALB/c dan FVB/J), and resistant (e.g., AKR/J, 129/svJ dan C57BL/6 J)^13^. In this study, we characterized the PM and M stages of lung SCC using NTCU in BALB/c mice which were categorized as the intermediate strain. Our study was the first to determine the optimal treatment duration for inducing PM and M lung SCCs i.e., 15 and 30 weeks, respectively. Briefly, a mixture of hyperplastic, metaplastic, and dysplastic lesions was observed after 15 weeks of NTCU treatment. Together they constitute the PM stage of lung SCC, since the epithelial cells in these lesions were found in prior studies to harbour genetic abnormalities driving the growth of malignant or cancer cells^27–29^. A mixture of SCC in situ and invasive SCC was observed after 30 weeks of NTCU treatment. They were classified as the M stage because the cancer cells have the potential to invade the surrounding stroma in SCC in situ and metastasize in invasive SCC^29^.

PM lung SCC has been successfully induced in several prior studies^13,18–20^. The earliest is by Wang et al. which reported that hyperplastic lesions formed in all categories of mouse strains after 32 weeks of treatment^13^. However, the exact treatment duration for inducing PM lung SCC was not determined. Another study was conducted to evaluate the effect of different durations of NTCU treatments (4, 8, 12, 16, 25, and 32 weeks) on the types of lung SCC lesions^18^. It was found that bronchial dysplastic lesions only formed after 25 weeks of NTCU treatment, which is inconsistent with our observation that bronchial dysplasia was induced after 15 weeks of NTCU treatment. It is worth noting that the study by Ghosh et al. used a lower NTCU dose of 0.02 M, as compared to 0.04 M used in our study. This may explain the longer time required to induce bronchial dysplasia, although the same intermediate strain was used which is FVB/n mice^13,18^. Nevertheless, our tested duration to induce PM stage; 15 weeks was fitted in the range of duration treatment tested by Riolobos et al. which observed hyperplastic lesions after 8 weeks of NTCU treatment and a combination of high-grade hyperplasia, metaplasia, and high-grade dysplasia after 24 weeks of NTCU treatment in FVB/n mice^20^. We also showed that 30 weeks of treatment was sufficient to induce advanced lung SCC in BALB/c mice, rather than the 32 weeks used by Wang et al.^13^. This improvement in shortening the period of carcinogen exposure in experimental animals is crucial to minimize the adverse effects caused by the carcinogen. Notably, some studies reported successful induction of lung SCC after 20 weeks^21^ and 28 weeks of NTCU treatment^17^. However, the susceptible mouse strains, namely A/J and NIH Swiss, were used in these studies, which may explain the shorter time needed to induce lung SCC.

Additionally, we found that NTCU induced lung SCC through progressive transformations of normal cells in a sequence similar to the pathogenesis of human lung SCC i.e., ‘hyperplasia-metaplasia-dysplasia-SCC’. Our findings are in accordance with a study conducted by Wang et al., who also observed the same sequence of transformations leading to lung SCC lesions in NTCU-treated A/J mice^5^, confirming the reproducibility and reliability of NTCU-based protocols in inducing lung SCC that closely mimics human lung SCC. Another point worth considering is the dose-dependent action of NTCU. We applied 25 µL of 0.04 M NTCU twice a week to induce lung SCC, as similar dosage was used in previous studies to induce advanced lung SCC in an intermediate strain of mice after 32 weeks of treatment^13,14,20^. Notably, a lower concentration of 0.02 M was found to yield variable success rates in inducing lung SCC in FVB/n mice. Ghosh et al. found that a concentration of 0.02 M effectively induced lung SCC^18^; but the same concentration and period of treatment was reported by Riolobos et al. to have failed to induce lung SCC^20^. In some studies, substantially lower concentrations of 0.008 M and 0.004 M were tested and found to be unable to induce lung SCC after 32 weeks^14^. Considering the findings of these studies, we suggest that 0.04 M is the optimum NTCU concentration for inducing lung SCC in BALB/c mice.

Because most lung SCC originate in the bronchi epithelial cells, epithelium thickness assessment has become one of the grading criteria for pulmonary lesions of lung SCC^30,31^. Epithelium thickness marks the progression of lung SCC growth, as evaluated in this study. We found that the bronchial epithelium thickened in the cancer group at both the PM and M stages, as the cells proliferated uncontrollably, resulting in the formation of more than one layer of surface bronchial epithelium, a condition known as hyperplasia. The thickness significantly increased from the PM to the M stage as cell proliferation continued to exceed cell apoptosis^32,33^. In fact, increased proliferation of bronchial epithelium was observed in NTCU-induced lung SCC in vivo^16,18^. Our findings are in line with a study by Surien et al., who found increased epithelium thickness in BALB/c mice after NTCU treatment^15^. For the analysis, we only measured the epithelium thickness of normal to SCC in situ stages as invasive SCC lacks intact basement membranes and cellular organisation, which are both required for epithelium thickness measurement. Based on our findings from the analysis of histopathology and epithelium thickness, we suggest that the vehicle group, which received 70% acetone alone, is adequate as a valid control group because it does not differ significantly from the control group which received normal saline in histological characteristics, histopathology scores, and epithelium thickness. Moreover, the 70% acetone used in the vehicle group had no adverse effects on the mice, as observed in the control group, which received normal saline.

In assessing the histological features of SCC, the formation of intracellular bridges alongside other SCC characteristics, particularly keratin pearls were evaluated. In this study, we found no keratin pearls in the M stage of lung tissue, indicating that our lung SCC model was poorly differentiated. Therefore, immunohistochemistry (IHC) staining was used to confirm the lung SCC subtype. According to the 2015 World Health Organization (WHO) classification of lung tumors, immunohistochemistry (IHC) was recommended as the first guideline for classifying lung cancer^34^. These additional assessments are required to increase the accuracy of lung cancer classification and, as a result, maximize therapeutic benefit as treatments differ depending on the type of lung cancer^35^. Cytokeratin 5/6 (CK 5/6), P40, and P63 are the most commonly used biomarkers to confirm the squamous lineage or lung SCC. These biomarkers are highly expressed in SCC and have been studied extensively^13,14,21^. Figure 5 shows that positive CK 5/6 protein expression in lung tissues treated with NTCU for 30 weeks confirmed the lung SCC subtype. CK5/6 is a large molecular weight cytokeratin and is expressed in cancers of epithelial origin, such as oesophageal, cervix, and lung SCC^36–39^. The protein was found to be expressed in 91.7% of primary and 100% of metastatic lung SCC^40^. The adenocarcinoma subtype of lung cancer also expressed CK 5/6 but to an appreciably lesser extent than lung SCC; 22.2% of primary adenocarcinomas and 15.4% of metastatic adenocarcinomas have been found to express CK5/6^40^.

Despite its usefulness, this model has a few limitations. The extended treatment duration required to induce lung SCC could result in toxicity, skin inflammation, and reduced gain in body weight. Furthermore, NTCU has been shown to elicit heterogeneous responses depending on the mouse strain. Therefore, the optimal NTCU dosage may vary with the mouse strain used, the research aims, and the experimental designs. Pilot studies may be necessary for new regimens of NTCU treatments to ensure maximum efficacy and reproducibility in inducing SCC lesions in the selected strain of mice.

## Conclusion

We discovered NTCU to be a potent chemical capable of inducing lung SCC through multiple steps which allowed for pre-malignant and malignant characterization to be evaluated in BALB/c mice. The malignant stage lung tissue was positive for CK 5/6 which indicated an SCC subtype of lung cancer. Although the NTCU-induced lung SCC mouse models still fall short of accurately replicating the complexity of human lung cancer, the high histopathology similarity suggests that this model is the best at mimicking human lung SCC disease and could be a useful tool to increase our understanding of the disease. From this study also, we suggested that normal saline be excluded as a control group since the acetone-treated group or vehicle group alone is adequate as a control group, reducing the number of animals involved in research. Our suggestion is to uphold the 3Rs principle of animal study, which are reduction, replacement, and refinement.

## Materials and methods

### Animals

All procedures involving the use of animals were subjected to approval from Universiti Kebangsaan Malaysia Animal Ethics Committee (UKMAEC) (FSK/2017/FATHIAH/24-MAY/846-MAY-2017-MAY-2020). All experimental procedures involving animal were conducted in accordance with the UKMAEC guidelines. This study was also carried out in compliance with the ARRIVE guidelines. Five weeks old female BALB/c mice with an average weight of 12–16 g were obtained from the Faculty of Veterinary Medicine, Universiti Putra Malaysia, and were allowed to adapt to laboratory conditions for two weeks with ad libitum access to water and normal mice diet before the experiment started. Throughout the experiment, all mice were housed under the same laboratory conditions of ambient room temperature and lighting (12 h light–dark cycle). The mice were randomly allotted into two main groups; pre-malignant (PM) and malignant (M). Then, the mice were further assigned into three groups with n = 6 per group: control (received normal saline), vehicle (received 70% acetone), and cancer group (received 25 µL of 0.04 NTCU). The treatment was given twice a week with an interval of 3.5 days for 15 weeks for PM stage groups and 30 weeks for M stage groups via skin painting at the dorsal back between shoulder blades. All mice were euthanized by an overdose of ketamine-xylazine and cervical dislocation at the end of the experiment.

### Hematoxylin & Eosin (H&E) staining

The harvested lung tissues were immediately washed with phosphate buffer saline (Sigma-Aldrich, USA) before being fixed in 10% formalin (Bendosen, Malaysia) for 24 h. Then, the tissues were processed using a tissue processor (Leica TP1020, Germany) and embedded in paraffin (Thermo Fisher Scientific, USA) using a tissue embedder (Leica EG1160, Germany). The tissues were sectioned into 4 µm of thickness using a microtome (Leica RM2135, Germany) from the centre area as lung SCC usually arises centrally. The sectioning was also performed perpendicularly to the bronchi divergence and one section was selected consistently in every five sections (approximately 20 µm apart). After that, the tissues were deparaffinized with xylene (Thermo Fisher Scientific, USA), and rehydrated in graded alcohol (HmbG Chemicals, Malaysia). For H&E staining, the tissues were then stained with hematoxylin (Leica Biosystem, USA), eosin (R&M Chemicals, Malaysia), and dehydrated with alcohol (HmbG Chemicals, Malaysia) and xylene (Thermo Fisher Scientific, USA) before being mounted with DPX (R&M Chemicals, Malaysia). Tissues were viewed using a light microscope (Nikon Corporation, Japan), and histological scoring of lung SCC was performed with an assistant of a veterinary pathologist. Three different sections of tissues were examined per mouse for histological scoring. The scoring was recorded as follow: normal bronchus = 0, presents with single-layer columnar epithelium; hyperplasia = 1, presents with more than one layer of the columnar epithelium; metaplasia = 2, presents with a combination of columnar and squamous epithelium while maintaining hyperplastic characteristic; dysplasia = 3, presents with disorientation, hyperchromatic and a high degree of nuclear polymorphism cells while maintaining hyperplastic and metaplastic characteristic; SCC in situ /invasive SCC = 4, presents with high nuclear to cytoplasmic ratio cells, intracellular bridge, invasive cancer out of basement membrane^15^. Similarly, epithelium thickness was also measured from three different sections per mouse using ImageJ software (Bethesda, Maryland, USA). Three different airways were chosen from a single section. The epithelium thickness was then measured at three different adjacent areas from a single airway. In brief, the average of epithelium thickness obtained from a mouse was calculated from the cumulative mean of three different areas per airway and three different airways per section.

### Immunohistochemistry (IHC) staining

Paraffin-embedded samples were sectioned to 4 µm sections on the charged slide (Porlab Scientific, China) and dried overnight at room temperature. After that, the tissues were deparaffinized in an oven at 60 °C and transferred in xylene for complete removal of paraffin. Then, the tissues were rehydrated in absolute alcohol and incubated with antigen retrieval solution (1 × 10 mM sodium citrate buffer) (Merck, Germany). After that, endogenous peroxidase activity was blocked with 3% H_2_O_2_ (Merck, Germany) for 10 min, and non-specific antibody binding sites were then blocked with 10% normal goat serum (NGS) (Dako, Denmark) for 10 min. The tissues were then incubated with CK 5/6 antibody (diluted to 1:150; Catalog No. PA5-1748; Cell signaling, USA) overnight at 4 °C and incubated with goat anti-rabbit horseradish peroxidase (diluted to 1:100; Catalog No. PI-1000; Vector Laboratories, USA) for 1 h at room temperature. Finally, the reactions were developed using the 3,3′-Diaminobenzidine (DAB) (Dako, Denmark) and counterstaining was performed with hematoxylin. Negative control tissues were incubated with 10% NGS without primary antibody. Semi-quantitative analysis of total pixel intensity of positive DAB brown staining was analyzed using ImageJ FIJI software (Java 8 version 64-bit).

### Statistical analysis

All data are presented as mean ± SEM of at least three biological replicates and analyzed using GraphPad Prism version 8.3.0. The normality test was carried out using Shapiro–Wilk. The differences between groups were analyzed using one-way ANOVA and student’s t-test. Statistical significance was considered when *P* < 0.05.
